# A Hybrid Approach for Image Acquisition Methods Based on Feature-Based Image Registration

**DOI:** 10.3390/jimaging10090228

**Published:** 2024-09-14

**Authors:** Anchal Kumawat, Sucheta Panda, Vassilis C. Gerogiannis, Andreas Kanavos, Biswaranjan Acharya, Stella Manika

**Affiliations:** 1 Department of Computer Science Engineering and Application, Sambalpur University Institute of Information Technology (SUIIT), Burla, Sambalpur 768018, India; akumawat_phdca@vssut.ac.in; 2 School of Computer Sciences, Veer Surendra Sai University of Technology (VSSUT), Burla, Sambalpur 768018, India; suchetapanda_mca@vssut.ac.in; 3 Department of Digital Systems, University of Thessaly, 41500 Larissa, Greece; vgerogian@uth.gr; 4 Department of Informatics, Ionian University, 49100 Corfu, Greece; akanavos@ionio.gr; 5 Department of Computer Engineering-AI, Marwadi University, Rajkot 360003, India; 6 Department of Planning and Regional Development, University of Thessaly, 38334 Volos, Greece

**Keywords:** image registration, feature detection, hybrid feature detector, rotation invariance, scale invariance, binary robust invariant scalable keypoints (BRISK), features from accelerated segment test (FAST), maximally stable extremal regions (MSER), oriented FAST and rotated BRIEF (ORB)

## Abstract

This paper presents a novel hybrid approach to feature detection designed specifically for enhancing Feature-Based Image Registration (FBIR). Through an extensive evaluation involving state-of-the-art feature detectors such as BRISK, FAST, ORB, Harris, MinEigen, and MSER, the proposed hybrid detector demonstrates superior performance in terms of keypoint detection accuracy and computational efficiency. Three image acquisition methods (i.e., rotation, scene-to-model, and scaling transformations) are considered in the comparison. Applied across a diverse set of remote-sensing images, the proposed hybrid approach has shown marked improvements in match points and match rates, proving its effectiveness in handling varied and complex imaging conditions typical in satellite and aerial imagery. The experimental results have consistently indicated that the hybrid detector outperforms conventional methods, establishing it as a valuable tool for advanced image registration tasks.

## 1. Introduction

Image registration is the process of aligning multiple scene images into a single, integrated image. This technique addresses common issues such as image rotation, scale, and skew, which often arise when overlaying multiple images. The primary goal of image registration is to automatically establish correspondence between different images, a crucial step for further processing in various applications [1]. These images may be acquired at different times, from different devices, or may produce different types of information. Image-registration methods can be classified into two main types: area-based approaches and feature-based approaches. Area-based methods compare intensity patterns in images through correlation matrices, while feature-based methods establish correspondence between different image features such as lines, points, and contours. Feature-based methods are generally more reliable than area-based methods but require complex calculations to establish correspondence between the source image and the target image.

The process of image registration has extensive applications in diverse fields such as medical imaging, remote sensing, and computer vision. In medical imaging, for example, image registration is critical for combining data from different imaging modalities (such as CT and MRI) to provide comprehensive information about a patient’s anatomy. In remote sensing, it enables the integration of images taken from different sensors and at different times to monitor environmental changes. In computer vision, it is essential for tasks such as object recognition and 3D reconstruction. The robustness and accuracy of the registration process directly impact the effectiveness of these applications, making the development of efficient registration algorithms a significant research focus [2].

Feature-Based Image Registration (FBIR) consists of four steps: feature detection, feature matching, transform model estimation, and image resampling and transformation. In the first step, features are detected in both the source and target images. These features can include regions, contours, edges, and corners [3]. In the feature-matching step, it is determined whether pixels from the source image correspond to pixels from the target image. If correspondence is established, matching is performed. In the transform model estimation step, a mapping function is built, with types and parameters estimated. Finally, in the image resampling and transformation step, the source image is transformed using the transform model.

The accuracy and efficiency of the feature-detection step are crucial as they directly influence the subsequent steps in the registration process. Several feature-detection algorithms have been developed, each with its own strengths and weaknesses. For example, Speeded Up Robust Features (SURF) and Scale-Invariant Feature Transform (SIFT) are known for their robustness but can be computationally expensive. Features from Accelerated Segment Test (FAST) is computationally efficient but lacks invariance to scale and rotation. Binary Robust Invariant Scalable Keypoints (BRISK) offers robustness to both scale and rotation, while Oriented FAST and Rotated BRIEF (ORB) provides a good balance between computational efficiency and robustness. Other algorithms like MinEigen and Maximally Stable Extremal Regions (MSER) focus on specific aspects such as stability and sensitivity to feature regions [4].

Here, we briefly define several pivotal terms to aid understanding of feature-based image registration: Robustness refers to the ability of a feature-detection algorithm to deliver consistent results under varying conditions such as noise, illumination changes, and occlusion. Invariance to scale and rotation describes the capability of an algorithm to identify features correctly regardless of image scaling and rotation. Stability indicates the consistency of feature detection across different images or different instances of the same scene. Sensitivity to feature region measures the degree to which an algorithm can detect subtle changes or small features within an image. These definitions provide a foundation for discussing the strengths and limitations of various algorithms used in the field.

In the realm of digital image processing, image registration is a critical task that involves aligning two or more images—often from different sensors, times, or viewpoints—into a single, cohesive framework. This process is fundamental in applications ranging from satellite imagery analysis to medical imaging and automated surveillance systems. The primary challenges in image registration include ensuring high accuracy in matching diverse images, reducing the computational time required to process these images, and addressing failures in registration due to complex image transformations. This study focuses on innovating feature-detection techniques that enhance the precision and efficiency of image registration, particularly tackling the computational demands and robustness against image variations.

To overcome the limitations of existing algorithms, we propose a novel hybrid feature-detection algorithm that combines the strengths of BRISK and FAST [5,6]. This hybrid algorithm aims to reduce the time required for feature detection while maintaining robustness to scale and rotation. By leveraging the complementary strengths of BRISK and FAST, the hybrid algorithm can provide more reliable and efficient feature detection, which is essential for accurate image registration.

This study introduces a novel hybrid feature detector that integrates the robustness of BRISK with the speed of FAST, addressing common limitations found in traditional methods. Unlike conventional detectors, our hybrid approach is designed to provide high accuracy and efficiency under diverse operational conditions, offering significant improvements in both scale and rotation invariance.

The remainder of this paper is structured as follows: Section 2 reviews related work, providing a critical examination of previous studies and developments in feature-detection techniques and their applications in image registration. Section 3 details the methodology, including the development and implementation of the hybrid feature-detection algorithm, and describes the image acquisition methods used. In Section 4, we present the simulation setup and discuss the results obtained from testing the proposed algorithm against established detectors, demonstrating its effectiveness through various performance metrics. Finally, Section 5 summarizes the findings and contributions of this study and outlines potential avenues for future research, highlighting opportunities for further enhancements and applications of the proposed hybrid feature detector.

## 2. Related Work

Image registration is a fundamental and crucial task in image processing, utilized to match two or more images acquired at different times, from different sensors, or from different viewpoints [7,8]. It plays an important role in integrating and analyzing images from various sources. Both classic and recent image-registration methods have been extensively reviewed, highlighting the advantages, drawbacks, and future research directions [9].

A comparative study of well-known feature detectors and descriptors, including SIFT, MSER with SIFT, SURF with SURF, BRISK with BRISK, FAST with BRIEF, and ORB with ORB, has been conducted [10]. Additionally, the performance of feature descriptors extracted by the Harris–Affine detector has been compared [11].

These methods often face challenges such as computational complexity, sensitivity to changes in lighting and scale, and robustness against noise and occlusion. Robustness refers to a method’s ability to deliver consistent performance under varying conditions, while scale-invariance denotes the capability of a method to handle images of different sizes and orientations effectively. Stability, another crucial term, indicates the consistency of a method in detecting features across similar or varying scenes.

Furthermore, the existing algorithms often exhibit limitations in terms of their adaptability to different application contexts. For instance, while algorithms like SIFT and SURF provide excellent feature detection and matching under uniform lighting conditions, they may underperform in scenarios with variable lighting or when capturing images from rapidly moving objects. This variability demands a nuanced understanding of each algorithm’s operational environment to optimize performance effectively. Moreover, the trade-off between computational demand and accuracy is a critical consideration, particularly in real-time applications where processing speed is paramount.

In response to the limitations of existing feature detectors, our research introduces a novel hybrid algorithm that combines the strengths of several well-established methods. This hybrid approach aims to mitigate individual weaknesses and enhance overall performance, particularly in challenging environments typical of remote sensing and automated surveillance. By leveraging composite techniques, the proposed method not only improves detection accuracy and speed but also enhances robustness against variations in lighting, scale, and movement, making it highly suitable for modern image registration demands.

A novel algorithm for multispectral facial recognition, incorporating both visible and infrared (IR) images using various feature detectors, has been proposed [12]. Multispectral image registration with scale-invariant feature transform (SIFT) and random sample consensus (RANSAC) were described in [13]. In another study, a feature-based image registration (FBIR) method using HOG (Histogram of Oriented Gradients) for keypoint matching and a six-parametric offline transformation model was introduced [14]. Registration methods and their challenges have been reviewed, with a performance evaluation based on registration accuracy [15].

Traditional and advanced methods for multimodal remote-sensing (MMRS) image registration algorithms have been discussed [16]. An enhanced affine transformation (EAT) algorithm for non-rigid IR and visible (VIS) image registration has been presented [17]. A novel spatially invariant feature-matching scheme with higher performance using similarity matrices based on normalized eigenvector correlation and signal directional differences has been proposed [18].

Various algorithms for feature detection and description have been investigated [19]. A new feature descriptor, histogram of angle, and maximal edge orientation distribution (HAED), has been developed to address multi-source image-matching problems [20]. A comprehensive survey on different feature-based image-matching procedures and methods has been conducted [21].

An image-matching algorithm known as Dominant Orientation of Gradient (DOG) has been found to be robust to nonlinear intensity variations [22]. A novel local statistics-based image registration scheme, robust to contrast changes and geometric transformations, has been introduced [23]. An intelligent framework using a hybrid structural feature extraction technique for estimating transformation parameters using ground truth images has been proposed [24]. Authors in [25] focused on real-time image registration for an accurate geographic position using a UAV aerial view of images.

A hybrid feature extraction technique for medical images has been presented [26], along with a robust coarse-to-fine registration (CCFR) algorithm [27]. A feature matching algorithm combining FAST feature points and SURF descriptors has been proposed [28]. To address the difficulty of accurately registering low-texture images, a high-precision image registration algorithm based on line segment features has been developed [29].

An optimization algorithm called the normal vibration distribution search-based differential evolution algorithm (NVSA) has been introduced for SAR and optical image registration [30,31]. The Efficient Attention Pyramid Transformer (EAPT) has been proposed to address the problem of patch detection, using deformable attention, encode-decode communication modules, and position encoding for patches of any dimension [32].

A combination of traditional machine learning algorithms and deep neural networks for feature extraction in color and space aspects has been utilized to develop a one-stop deep portrait photographing guidance system [33]. A temporally broad learning system (TBLS) has been proposed to maintain temporal consistency between frames, consisting of original frames and corresponding frames in temporally inconsistent videos [34].

A novel deep convolutional neural network utilizing the multimodal cascaded method for detecting and classifying domestic waste has been proposed, along with a smart trash bin (STB) as the front-end carrier for waste disposal [35]. A Generative Parking Spot Detection (GPSD) algorithm using corner points to recover parking spots has been developed, featuring a layered analytical illumination balance method and a fast micro-target detection network [36]. Lastly, a broad attentive graph fusion network (BaGFN) has been designed to strengthen high-order feature representation under graph structures and refine high-order feature interactions at a bitwise level [37].

In the realm of image processing, the selection of an appropriate feature-detection and registration method is pivotal for achieving high accuracy and efficiency. Table 1 provides a succinct overview of various well-established and novel algorithms discussed in the literature. These methods are characterized by their unique attributes and are applied across a diverse array of scenarios.

## 3. Methodology

**Figure 1:**
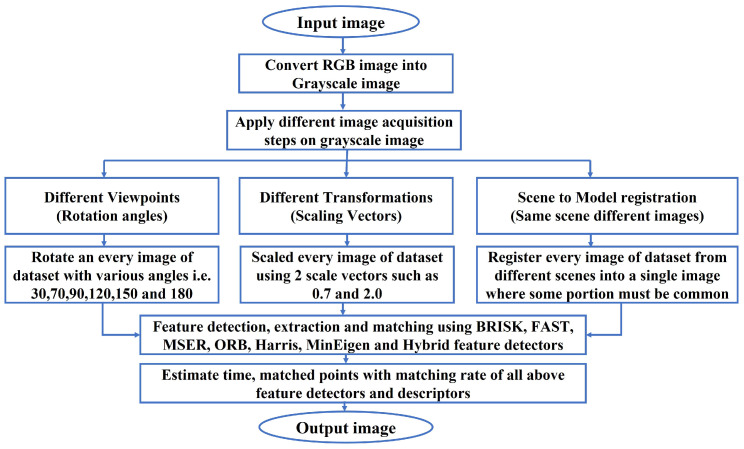
Flow diagram of the proposed methodology.

This paper proposes a novel algorithm resulting from a combination of the BRISK and FAST feature-detection algorithms. The proposed methodology is outlined in the flow diagram shown in Figure 1. Detailed explanations of all the feature detectors and descriptors used in this study are provided in the following section.

The experiments conducted in this study focus on three distinct types of image registrations based on the manner of image acquisition: (i) image registration based on different viewpoints with varying rotation angles, (ii) scene-to-model registration using two different scenes with some common portions, and (iii) image registration based on different viewpoints with scaling transformations. These specific registrations were chosen to methodically assess and demonstrate the capabilities of our hybrid algorithm under controlled variations.

By separating these transformations in the initial experiments, we aimed to isolate the effects of each type of manipulation on the detection performance, providing a clear understanding of how each adjustment affects the overall efficiency and accuracy of our proposed hybrid detector. This approach allows for a more granular analysis of performance under specific conditions, which is essential for developing a robust feature-detection system.

The registrations are performed on a diverse set of remote-sensing and scene images, encompassing a broad range of real-world scenarios to evaluate the performance of the proposed hybrid algorithm comprehensively. Future work may include combining these transformations to simulate more complex real-world scenarios, further testing the adaptability and robustness of the hybrid detector.

In our manuscript, we focus on empirical performance metrics such as matching rate, authentication rate, and computation time. These metrics are directly applicable to the practical deployment of image registration techniques and provide clear, measurable outcomes that can be compared across different methods; while theoretical frameworks involving cost function minimization are valuable for certain analytical or optimization-focused studies, our approach prioritizes direct evaluation of the methods in terms of their operational effectiveness in real-world scenarios. This choice is aligned with the needs of applications that require fast and accurate feature detection and registration, such as remote sensing and automated surveillance.

### 3.1. Feature Detectors and Descriptors

A novel feature detector for keypoint detection, description, and matching known as BRISK (Binary Robust Invariant Scalable Keypoints) has been proposed [40]. This method is recognized for its robustness to scale and rotation, making it suitable for various image-registration tasks. BRISK (Binary Robust Invariant Scalable Keypoints) provides a fast and robust solution for keypoint detection and description, ideal for real-time applications. It creates scale–space pyramids to achieve scale invariance and uses a pattern-based descriptor for robustness against rotation. However, it may produce higher false positive rates compared to slower, more complex methods like SIFT.

FAST (Features from Accelerated Segment Test) employs machine learning algorithms to enhance the efficiency of feature detection [39]. A subsequent work presents a heuristic approach for feature detection, further improved by machine learning techniques [41], significantly enhancing the speed and performance of the algorithm. FAST is renowned for its computational speed by using a decision tree to quickly assess pixel intensities. Although highly efficient, FAST lacks rotational invariance and is not inherently scale-invariant, which may limit its application in environments where orientation and scale vary significantly. Extensions like ORB have been developed to incorporate scale and rotation invariance into FAST.

ORB (Oriented FAST and Rotated BRIEF) builds on FAST by adding a pyramid scheme for scale invariance and a learning-based orientation mechanism. ORB is partially scale-invariant and more robust to rotation variations compared to FAST alone, making it a versatile choice for multi-scale, orientation-varied feature-detection tasks. However, it may still struggle with high levels of image noise and significant scale changes.

In a comprehensive comparison of feature detectors such as SIFT, ORB, AKAZE, BRISK, MinEigen, and SURF, SIFT and BRISK have been identified as more accurate, while ORB and BRISK demonstrate higher efficiency [42,43]. Additionally, SURF, BRISK, and SIFT are noted for their scale-invariance, with ORB being less so. This evaluation underscores the varying capabilities and suitability of these algorithms for different image-registration tasks, emphasizing the need for a hybrid approach to combine their strengths.

The MSER (Maximally Stable Extremal Region) detector, which is known for its robustness and efficiency, utilizes the component tree as an efficient data structure, allowing quasi-linear time calculation of MSERs [38]. This feature detector is particularly useful for tracking applications due to its stability. MSER detects regions in an image that are stable and distinctive. Stability ensures that regions remain consistent under slight image perturbations such as noise or geometric transformations, while distinctiveness means regions visually stand out from their surroundings. This makes MSER particularly effective for identifying text or other significant structures within varied imaging conditions. It analyzes intensity variations, identifying connected areas with similar intensity that maintain their shape and intensity over transformations, making it suitable for applications like text recognition and object segmentation. Despite its robustness, MSER’s performance may vary depending on image contrast and noise, sometimes requiring combination with other techniques for optimal results.

The Harris corner detector examines the eigenvalues of the autocorrelation matrix to detect corners and interest points in images [44]. This method is well regarded for its precision in identifying distinct image features.

By integrating the strengths of BRISK and FAST, the proposed hybrid algorithm aims to achieve efficient and robust feature detection suitable for a wide range of image-registration tasks. The proposed hybrid detector combines the high-speed processing of FAST with the scale and rotation invariance of BRISK, enhancing both the accuracy and the efficiency of keypoint detection. This improvement is particularly beneficial in complex image transformations and diverse operational scenarios, as detailed in the subsequent sections on methodology and simulation results.

Moreover, the application of the hybrid detector in challenging environments, such as remote sensing and automated surveillance, has demonstrated superior performance in terms of both detection accuracy and computational efficiency. The hybrid approach effectively addresses the limitations of individual feature detectors by dynamically adjusting to the characteristics of the input images and the specific requirements of the task. This adaptability is crucial in environments where image quality and scene complexity can vary significantly, thus requiring a more robust and flexible approach to feature detection.

#### Hybrid Feature-Detection Technique

The proposed hybrid approach combines the strengths of both the BRISK and FAST algorithms to address their individual limitations and enhance the overall performance of feature detection. This innovative technique leverages a ‘diagonal strategy’ within a circle of sixteen pixels for efficient corner detection, focusing on only four key pixels. This method not only speeds up the detection process but also maintains high accuracy by categorizing pixels into brighter, darker, and similar sections based on their intensity.

By integrating the detailed, scale-invariant detection capabilities of BRISK with the high-speed, efficient processing of FAST, the hybrid method significantly reduces both time and computational complexity. This dual approach ensures that the hybrid detector can quickly and accurately process images, making it especially suitable for complex image transformations and diverse operational scenarios found in remote sensing and automated surveillance.

Furthermore, the adaptability of the hybrid detector to varying imaging conditions—owing to its combined algorithmic structure—provides enhanced robustness against changes in lighting, scale, and motion. This robust performance is crucial for applications requiring reliable and precise feature detection in dynamic environments.

### 3.2. Feature-Based Image Registration (FBIR)

The problem of image registration, particularly FBIR, is a crucial issue in the image processing and computer vision domains. FBIR is mainly applicable in scenarios where it is necessary to integrate and analyze information from different sources, which may include different sensors, multiple photographs, various times, depths, or viewpoints. The diverse application areas of FBIR include image fusion, change detection, and multi-channel image restoration.

#### 3.2.1. Feature Detection and Extraction Using Proposed Hybrid Feature Detector

The first step of FBIR involves feature detection and extraction. An automatic machine-based method is developed to extract structures and features from images. Features can be regions like forests, lakes, and fields, or points such as region corners and line intersections.

An automatic hybrid approach for feature detection and description is developed to address the limitations of the BRISK and FAST algorithms [5]. The proposed hybrid detector and descriptor aim to reduce both time complexity and computational complexity, and while the BRISK algorithm is robust to rotation and scale, it is computationally intensive. Conversely, the FAST algorithm is faster but not scale-invariant and depends on a threshold value. By combining the advantages of both BRISK and FAST, the proposed approach overcomes their respective drawbacks.

In the proposed hybrid feature detector and descriptor, a corner pixel is constructed by considering a circle of sixteen pixels. These pixels are divided into three groups: brighter, darker, and similar portions, identified by their threshold and intensity values. Suppose the threshold value is represented by *t* and the intensity of the pixel by *i*.

(1) $$\mathrm{Intensity}\;\mathrm{of}\;a\;\mathrm{pixel}=\left\{\begin{array}{cc} t+i & \mathrm{if}\;\mathrm{brighter}\\ t-i & \mathrm{if}\;\mathrm{darker}\\ t-i\leq i\leq t+i & \mathrm{if}\;\mathrm{similar} \end{array}\right.$$

For the brighter section, *i* is added to *t*; for the darker section, *i* is subtracted from *t*; and for the similar section, *i* lies between t−i and t+i. Using these three types of pixels, a diagonal approach is employed for the input sixteen pixels.

**Figure 2:**
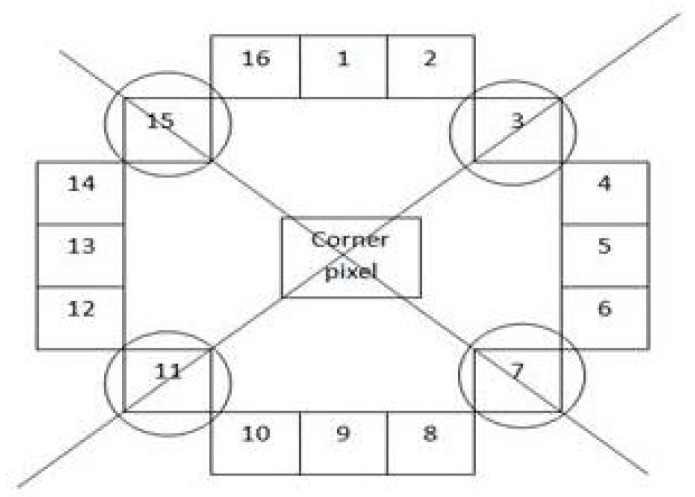
Diagonal approach for hybrid feature-detection method.

As shown in Figure 2, instead of testing all sixteen pixels, which is time consuming, the proposed hybrid algorithm tests only four pixels. This diagonal approach uses two slanting lines: one from pixel 3 to pixel 11 (right to left) and another from pixel 15 to pixel 7 (left to right). The intersection of these two diagonal lines identifies the center corner pixel. This method accelerates the process by reducing the number of comparisons. Finally, a local gradient method is applied to the center corner pixel to determine if the pixel’s distance is below or above the threshold value. The main advantage of the proposed algorithm is the reduction in both time and computational complexity compared to BRISK, FAST, MSER, MinEigen, ORB, and Harris algorithms. Additionally, the proposed algorithm outperforms existing algorithms in terms of match points and match rate.

Algorithm 1 outlines the steps employed in the Feature-Based Image Registration (FBIR) using the proposed hybrid feature detector and descriptor. The algorithm aims to optimize the feature-detection and matching process by applying various transformations and utilizing a hybrid approach that combines the strengths of BRISK and FAST algorithms. The result is an improved FBIR system that enhances the efficiency and accuracy of image registration, crucial for applications in areas like remote sensing, medical imaging, and computer vision.
**Algorithm 1** FBIR with Proposed Hybrid Algorithm.**Require:** Original image **Ensure:** Registered image using FBIR with hybrid feature detector and descriptor  1:Obtain the required input image from the database and convert it to its grayscale equivalent.  2:Apply transformations on the grayscale image:
For rotation of an image using different angles:
$$R\left(x^{\prime },y^{\prime }\right)=\left\{\begin{array}{c} x\cos\theta -y\sin\theta \\ x\sin\theta +y\cos\theta \end{array}\right.$$
where *R* is the rotated resultant image, $x^{\prime }$ and $y^{\prime }$ are the rotated pixel coordinates, and θ is the angle of rotation.For scaling transformation of an image:
$$S\left(x^{\prime },y^{\prime }\right)=\left\{\begin{array}{c} x\cdot s_{x}\\ y\cdot s_{y} \end{array}\right.$$
where *S* is the scaled image, $x^{\prime }$ and $y^{\prime }$ are the new scale coordinates, and $s_{x}$ and $s_{y}$ are the scaling factors.Perform scene-to-model description and detection using two different input images with some common portions.  3:Detect feature keypoints from both the reference image and the sensed image using various detectors like BRISK, FAST, MSER, ORB, MinEigen, Harris, and the proposed hybrid feature detector.  4:Extract features from both detected images using the transformations described in previous steps with various detectors and the proposed hybrid detector.  5:Match the key feature points extracted from both the reference image and the sensed image using affine transformation with bicubic/bilinear interpolation.  6:Estimate the time for rotation, scaling, and scene-to-model registration for all detectors, including the proposed hybrid detector.  7:If the matched points from both images are successfully extracted, obtain the registered image.


To further elucidate the operation of our proposed Hybrid Feature Detector, we utilize a strategic subset of pixels within a predefined circular pattern. Initially, all 16 pixels within this pattern are assessed based on their intensity values to classify them into three categories: brighter, darker, and similar. This categorization allows the algorithm to focus computational efforts on four pivotal pixels, which are determined through a diagonal evaluation approach. This method not only streamlines the feature-detection process by reducing unnecessary computations but also maintains high detection accuracy by focusing on the most informative pixels.

The hybrid detector is designed to integrate the robust detection capabilities of BRISK, which is adept at handling scale and rotation variations, with the computational efficiency of FAST. This integration addresses the time-consuming keypoint detection in BRISK and the scale limitations of FAST, providing a balanced solution that is both fast and scalable.

To aid in the understanding of this integration, the following Algorithm 2 illustrates the process of selecting key pixels and combining the strengths of BRISK and FAST in our hybrid detection approach:
**Algorithm 2** Hybrid Feature-Detection Process.**Require:** Image **Ensure:** Keypoints
  1:Start with a circle of 16 pixels around each candidate pixel.  2:Classify 16 pixels into three categories based on intensity:
BrighterDarkerSimilar  3:Apply a diagonal strategy to select 4 key pixels:
Draw two diagonal lines across the circle.Select the intersection points as key pixels.  4:Use BRISK for scale and rotation invariant detection on selected pixels.  5:Apply FAST for quick detection on the reduced pixel set.  6:Combine results to obtain final keypoints.


This comprehensive approach ensures that our hybrid detector not only optimizes computational resources but also adapts dynamically to various imaging conditions, thereby enhancing both the performance and applicability of the feature-detection process in real-world scenarios.

#### 3.2.2. Feature Matching Using a Hybrid Algorithm

Feature matching, the second step of FBIR, plays a crucial role in establishing the mapping between two images of the same fields acquired from different sources. Keypoints are identified in both the reference image and the sensed image to perform matching. The goal is to find a better correspondence between these images by comparing each feature keypoint of the sensed image to those of the reference image and measuring the distance between these points using BRISK, FAST, and the hybrid feature descriptors. The Euclidean distance between two keypoints $\left(a_{1},b_{1}\right)$ and $\left(a_{2},b_{2}\right)$ is calculated using the following formula:

(2) $$\mathrm{distance}=\sqrt{{\left(a_{2}-a_{1}\right)}^{2}+{\left(b_{2}-b_{1}\right)}^{2}}$$

The distance is calculated for various feature detectors, including BRISK, FAST, MSER, MinEigen, ORB, Harris, and the hybrid algorithm. To compare the execution time of each algorithm, three time-based parameters are used: elapsed time, CPU time, and PMT time. Experimental results show that the proposed hybrid feature matching algorithm takes less time compared to existing feature detectors and descriptor algorithms.

#### 3.2.3. Feature-Based Transform Model Estimation

The third step of FBIR involves estimating both the type and parameters of the transformation needed to align the source image with the target image. The goal is to find an accurate transform model that appropriately transforms the source image. Three methods for parameter estimation in transform model estimation are discussed, with affine transformation being used in this paper.

Similarity transformation is a shape-preserving mapping transformation that preserves angles and curvatures, while affine transformation is a linear mapping method that preserves points, straight lines, and planes. Affine transformation is applied to correct geometric deformations that occur due to non-ideal camera angles, and it is a particular case of projective transformation.

#### 3.2.4. Image Resampling and Transformation

The final step of FBIR is image resampling, which involves changing the pixel dimensions of an image, effectively altering its resolution. The registered image obtained from the previous step is convolved with an interpolation kernel. Interpolation techniques reduce the bandwidth of the signal by employing a low-pass filter on the discrete signal.

Three interpolation techniques are compared: Nearest Neighbor, Bilinear, and Bicubic. These techniques are evaluated based on image quality parameters such as Mean Squared Error (MSE), Root Mean Squared Error (RMSE), Signal-to-Noise Ratio (SNR), and Peak Signal-to-Noise Ratio (PSNR), as shown in Table 2.

From Table 2, it can be observed that the MSE for Bicubic interpolation is 0.00214, 0.00219, and 0.00221 for affine, similarity, and projective transformations, respectively. These values indicate that Bicubic interpolation has the lowest error content compared to Bilinear and Nearest Neighbor interpolation schemes. Similar observations can be made for RMSE, SNR, and PSNR values, where Bicubic interpolation outperforms the other methods. Lower values of MSE and RMSE imply less error content, while higher values of SNR and PSNR indicate lower noise content in the image. Hence, Bicubic interpolation, which produces a smoother interpolation surface, is used in the image resampling and transformation step of image registration in this paper.

## 4. Simulation and Results

This section validates the proposed hybrid algorithm using a series of experiments conducted on eight aerial images. The validation considers three primary types of transformations: rotation at various angles, scene-to-model transformation using different instances of the same image where some parts share common features, and scaling transformations at varying scales.

The results of these experiments, presented in both numerical and visual formats, demonstrate that the proposed hybrid feature-detection algorithm outperforms existing detectors such as BRISK [40], FAST [41], ORB [43], Harris [44], MSER [38], and MinEigen [42]. Our method not only reduces the computational time required for feature detection but also improves the accuracy of keypoint matching, making it a valuable tool for a wide range of image registration applications.

To facilitate transparency and allow for in-depth validation by the research community, the complete source code used in our experiments is available on GitHub [45]. This includes the Matlab 2019a implementation of our algorithm to assist with setup and replication of our results.

### 4.1. Experimental Setup and Image Data

The experiments were conducted using eight different images from the AID (Aerial Image Dataset) database, representing a variety of scenes including parks, railway stations, airports, bridges, a university gate (VSSUT gate), and a large dam (Hirakud dam) [46]. These images were chosen to cover a broad spectrum of typical scenarios in remote sensing and feature-detection tasks.

These images are subjected to three types of transformations to test the robustness and effectiveness of the hybrid feature-detection algorithm:

- Rotation: Images are rotated at angles of $30^{\circ }$, $70^{\circ }$, $90^{\circ }$, $120^{\circ }$, $150^{\circ }$, and $180^{\circ }$.
- Scene-to-Model Transformation: This involves using two different instances of the same scene (e.g., different views of an airport and a bridge) where parts of these images share common features.
- Scaling: Images are scaled by factors of 0.7 and 2.0 to evaluate the algorithm’s performance under size variations.

**Figure 3:**
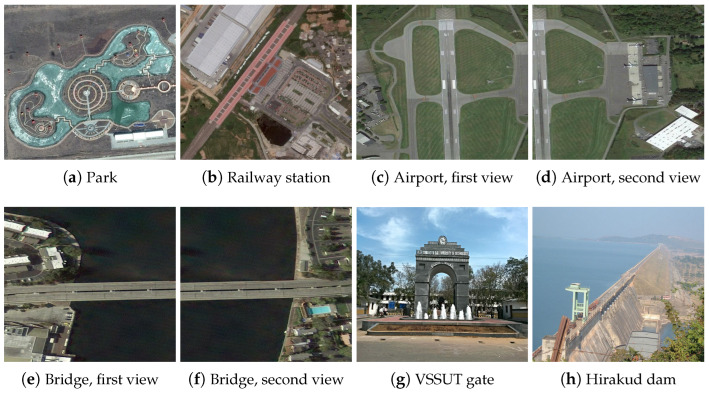
Sampled color images from AID database [46].

**Figure 4:**
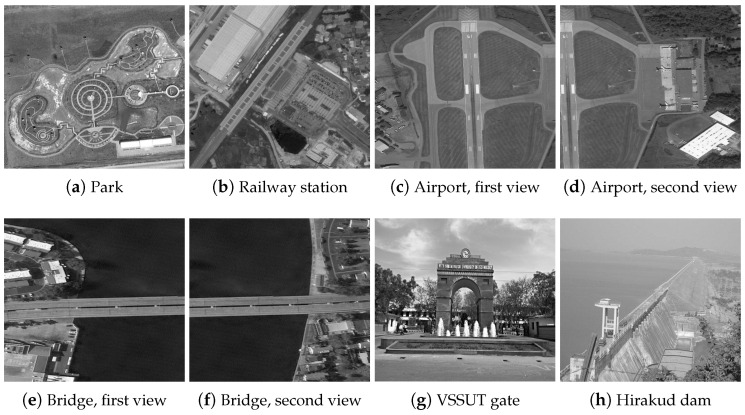
Grayscale conversion of sampled color images from AID database [46].

**Figure 5:**
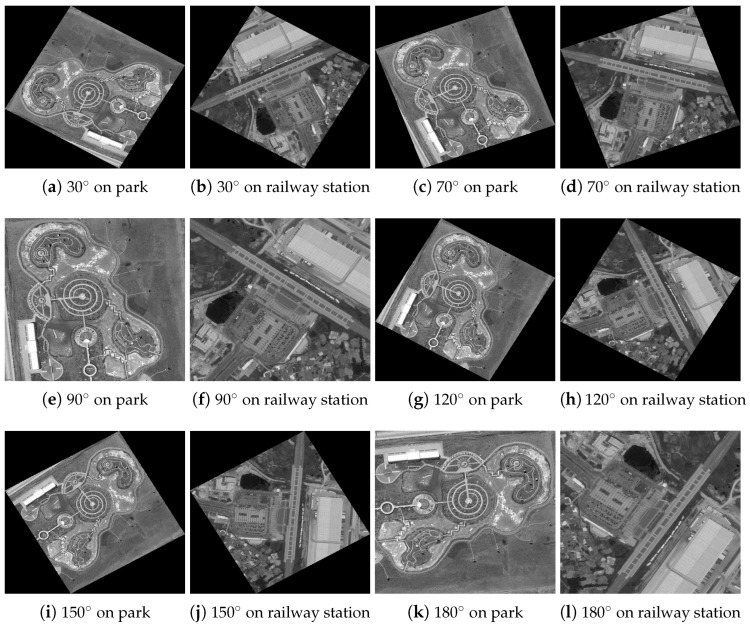
Various rotation angles applied on park and railway station grayscale aerial images.

**Figure 6:**
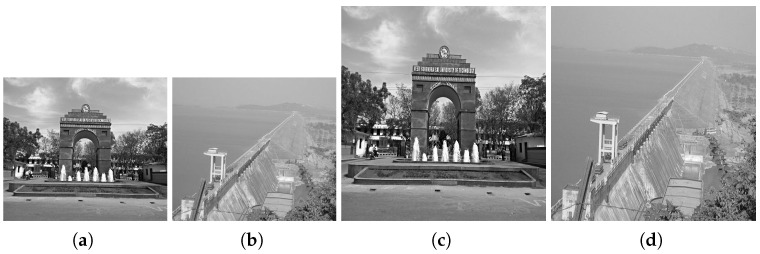
Scaling transformations applied to VSSUT gate and Hirakud dam images. (**a**) 0.7 scaling factor on VSSUT gate. (**b**) 0.7 scaling factor on Hirakud dam. (**c**) 2.0 scaling factor on VSSUT gate. (**d**) 2.0 scaling factor on Hirakud dam.

Figure 3 presents the original color aerial images from the AID database. Figure 4 shows these images converted to grayscale. Figure 5 illustrates the effects of various rotational angles applied to the park and railway station images. Figure 6 displays the scaling transformations applied to the VSSUT gate and Hirakud dam images.

#### 4.1.1. Time Measurement Definitions

In our experimental analysis, we utilize three primary metrics to evaluate the computational efficiency of the feature-detection algorithms:

- Elapsed Time: total time from the initiation to the completion of the feature-detection process.
- CPU Time: the amount of processing time the CPU spends to execute the feature-detection tasks, excluding any idle time.
- PMT (Performance Measuring Time): this metric assesses the performance efficiency of the algorithm, focusing on the active processing time.

These metrics help in understanding the computational demand and efficiency of the proposed methods under different operational conditions.

#### 4.1.2. Validation of Detected Keypoints

To ensure the accuracy of detected keypoints, our analysis relies on the established performance metrics such as precision and matching rate, which have been detailed in previous sections. These metrics serve as indicators of the correctness of the keypoint identification:

- Precision assesses the proportion of detected keypoints that are true positives, helping to confirm that the keypoints are genuine features of the images rather than noise or errors.
- Matching Rate evaluates how well the keypoints from different transformations of the same image correlate with each other. A high matching rate indicates a successful identification of consistent and reliable keypoints across different versions of the images.

This analytical approach allows us to validate the effectiveness of the keypoint detection algorithm without the need for additional experimental validation. The high performance metrics reported reflect the robustness of our feature-detection algorithm, underscoring its reliability in identifying correct keypoints even under challenging conditions such as rotation and scaling.

### 4.2. Rotation with Different Angles

This subsection meticulously evaluates the performance of various feature-detection algorithms under rotation transformations, focusing on their robustness and effectiveness. Two distinct aerial images—a serene park and a bustling railway station—serve as test subjects. These images were methodically rotated at six pivotal angles: $30^{\circ }$, $70^{\circ }$, $90^{\circ }$, $120^{\circ }$, $150^{\circ }$, and $180^{\circ }$. This setup aims to rigorously test the resilience of the feature-detection methods, including an innovative hybrid algorithm developed as part of this study.

Several renowned feature detectors were employed in these experiments: BRISK [40], FAST [41], ORB [43], Harris [44], MinEigen [42], and MSER [38], alongside the newly proposed hybrid detector. Each detector’s capability to consistently identify and track feature keypoints across various rotation angles was analyzed.

**Figure 7:**
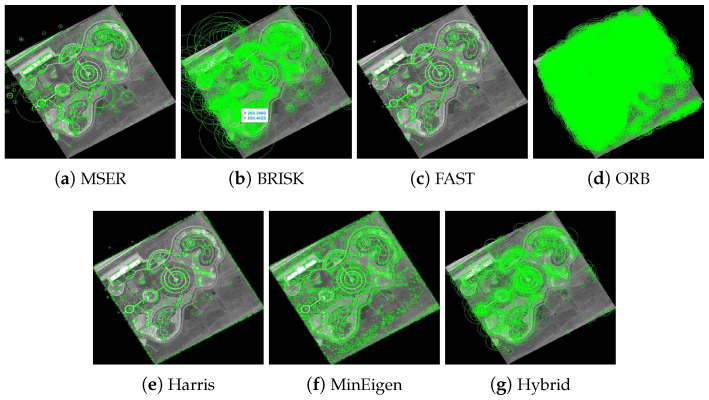
Detection of feature keypoints in the park image under $150^{\circ }$ rotation, showcasing the performance of different detectors. Green markers highlight the keypoints detected, with each subfigure corresponding to the output using a different feature-detection method.

**Figure 8:**
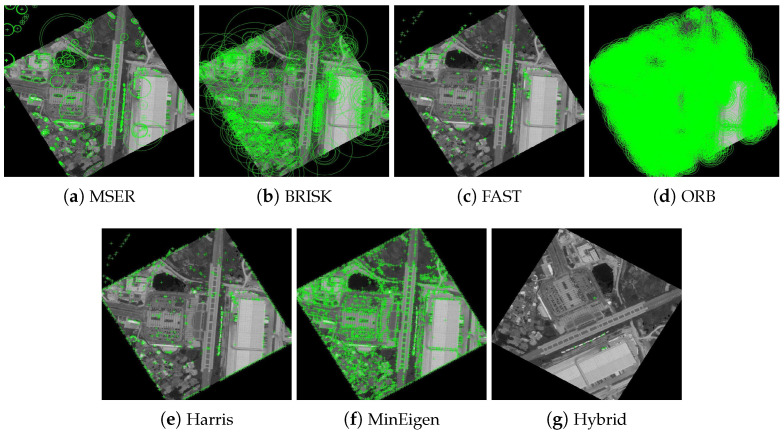
Detection of feature keypoints in the railway station image under $150^{\circ }$ rotation, showcasing the performance of different detectors. Green markers indicate the keypoints, and each subfigure corresponds to the output using a different feature-detection method.

The results of these experiments are illustrated in Figure 7 and Figure 8. These figures not only depict the detection of feature keypoints but also highlight the comparative performance and distinctive traits of each detector under rotational stress. Such detailed visualization aids in understanding the practical impacts of rotational transformations on feature-detection reliability. The feature keypoints, indicated in green, represent significant aspects of the image such as edges, corners, or other specific patterns that are crucial for alignment tasks in image registration. This detailed visualization helps to better understand how different detectors perform under the challenge of rotation, providing insight into their robustness and effectiveness.

**Figure 9:**
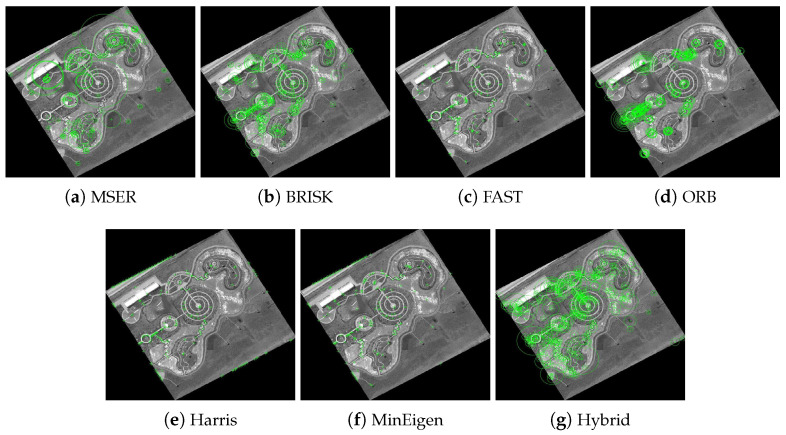
Extraction of feature keypoints from the park image under $150^{\circ }$ rotation. Green markers demonstrate the keypoints extracted, emphasizing the nuances of each algorithm with each subfigure showing results using a different feature extraction method.

**Figure 10:**
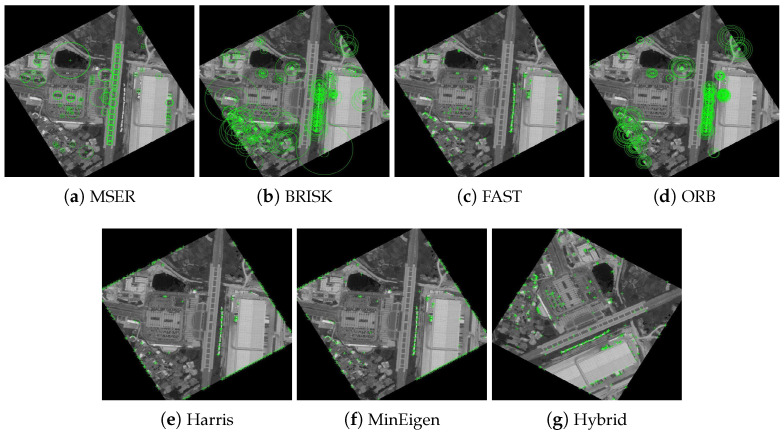
Extraction of feature keypoints from the railway station image under $150^{\circ }$ rotation. Each subfigure demonstrates the results using a different feature extraction method, with green markers used to emphasize keypoint locations and algorithmic nuances.

Subsequent Figure 9 and Figure 10 extend this analysis by detailing the extraction processes of these keypoints for both the park and railway images. The robust extraction capabilities of each feature detector are crucial for accurate feature matching in applications such as image stitching and object recognition in computer vision. The green markers in these figures specifically illustrate the keypoints that each detector has identified as crucial for successful image analysis and manipulation. This step-by-step visualization showcases the effectiveness of each algorithm in maintaining keypoint integrity even through complex transformations, ensuring accurate subsequent image registration.

**Figure 11:**
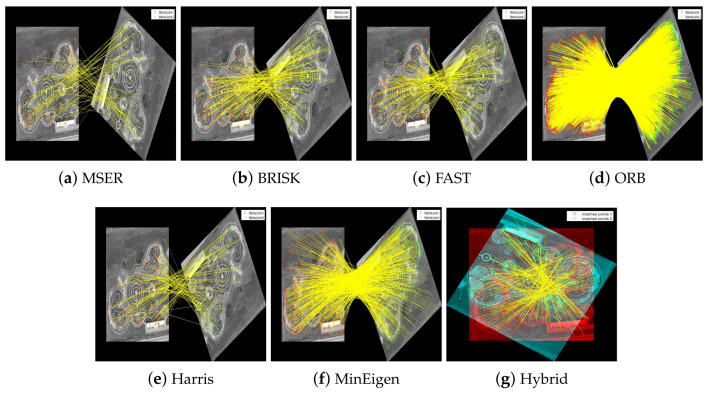
Matching of feature keypoints in the park image across different rotational views under $150^{\circ }$ rotation. Subfigures (**a**–**f**) display the matched keypoints separately to illustrate individual detector performance clearly. Subfigure (**g**) shows an overlaid result of the hybrid detector to demonstrate the integration of multiple detection outcomes, providing a comprehensive view of the keypoints matched by the proposed method. Each image aims to highlight the effectiveness of each feature detector in achieving consistent matching across transformations.

**Figure 12:**
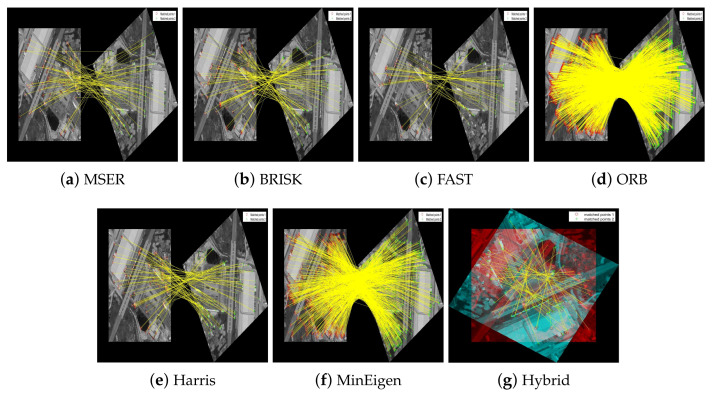
Matching of feature keypoints in the railway station image across different rotational views under $150^{\circ }$ rotation. Each subfigure highlights the effectiveness of each feature detector in achieving consistent matching.

In our evaluation of the feature detectors, we carefully analyze how each method performs under various transformations. Figure 11 and Figure 12 are designed to provide a clear comparison between the detectors. To make the differences more discernible, the images are presented separately for individual detectors to allow for a focused analysis of each method’s capabilities in isolation. For the hybrid detector, we present an overlaid image to demonstrate the synergistic effect of combining multiple detection techniques, showcasing our proposed method’s comprehensive matching capability. This format aids in visualizing the distinct performance traits and alignment precision of each detector.

In addition to visual assessments, the performance of the feature-detection algorithms was quantitatively evaluated, as depicted in Table 3 and Table 4. These tables enumerate the detected keypoints, extracted keypoints, and matched keypoints across six rotational angles for both the park and railway station images, employing a variety of feature detectors including BRISK, FAST, ORB, Harris, MinEigen, MSER, and the newly proposed hybrid detector.

The hybrid algorithm, notably, demonstrated superior performance in terms of match rate percentages, significantly outperforming traditional detectors. For example, at a $120^{\circ }$ rotation, the hybrid detector achieved a match rate of 28.84%, which is the highest among the detectors tested. Moreover, in scenarios with $90^{\circ }$ and $180^{\circ }$ rotations, the hybrid and ORB detectors achieved a perfect match rate of 100%, indicating robust performance in standard upright and inverted orientations.

Table 3 includes key statistical metrics—mean, variance, and standard deviation—across various rotation angles to provide a clearer, summarized view of the performance of different feature detectors. These statistical summaries help discern the general trends and variability in the performance of each method without the clutter of individual data points. For instance, at a $90^{\circ }$ rotation, the mean matching rate is notably high, reflecting the robustness of the detectors under orthogonal rotations. The standard deviation at this angle is lower compared to other angles, indicating more consistent performance across different detectors. This statistical approach not only simplifies the comparative analysis but also enhances the readability and interpretability of the results, supporting a stronger, more justified conclusion about the superiority of specific methods, such as the Hybrid detector which consistently shows high efficiency and accuracy.

The efficiency of the algorithms was also gauged through performance metrics such as elapsed time, CPU time, and PMT (Processor Memory Time). The hybrid algorithm consistently showed the lowest time consumption across these metrics, suggesting its suitability for real-time applications. For the park image at $150^{\circ }$ rotation, the hybrid algorithm required only 3.7125 s of CPU time, which is significantly less than the other detectors like BRISK (5.1875 s) and ORB (11.2813 s).

This numerical analysis confirms the effectiveness of the hybrid algorithm not only in maintaining high accuracy in feature matching across varied rotations but also in ensuring computational efficiency. Such attributes make the hybrid algorithm particularly advantageous for applications in remote sensing and medical imaging where rapid and reliable feature detection is critical.

### 4.3. Scene-to-Model Registration

Scene-to-model registration involves comparing different instances of the same image, typically referred to as the reference image and the sensed image, which share some common features. This process is crucial for applications such as satellite image analysis, where changes over time within the same geographic area need to be identified accurately. In this experiment, instances of aerial images from an airport and a bridge were selected to demonstrate the effectiveness of various feature-detection and matching algorithms.

**Figure 13:**
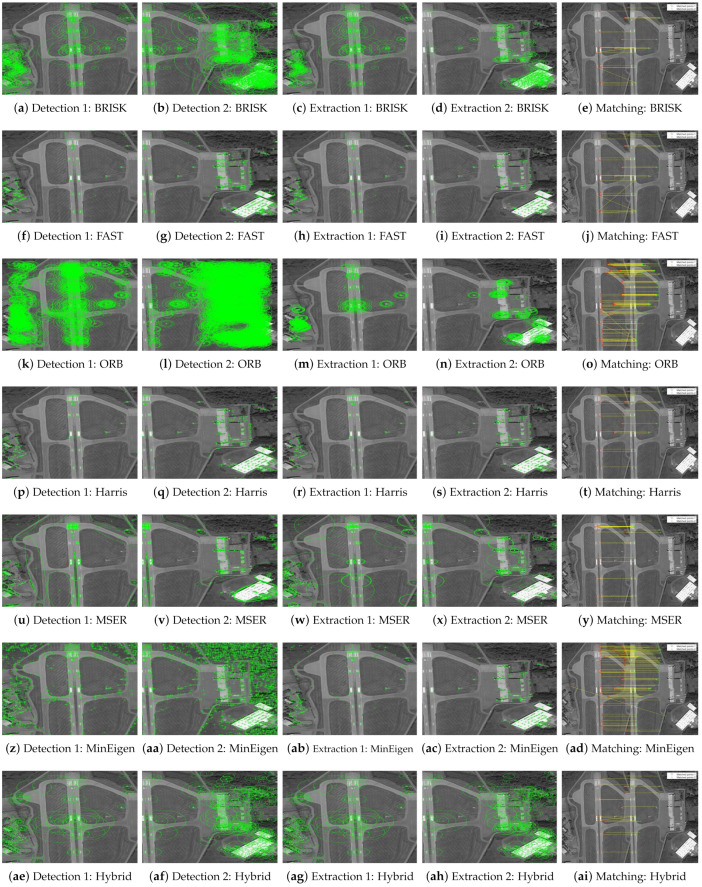
Sequential presentation of detection, extraction, and matching phases for various feature detectors on two sets of airport aerial images. Each row represents a different detector and showcases the process from detection to matching.

**Figure 14:**
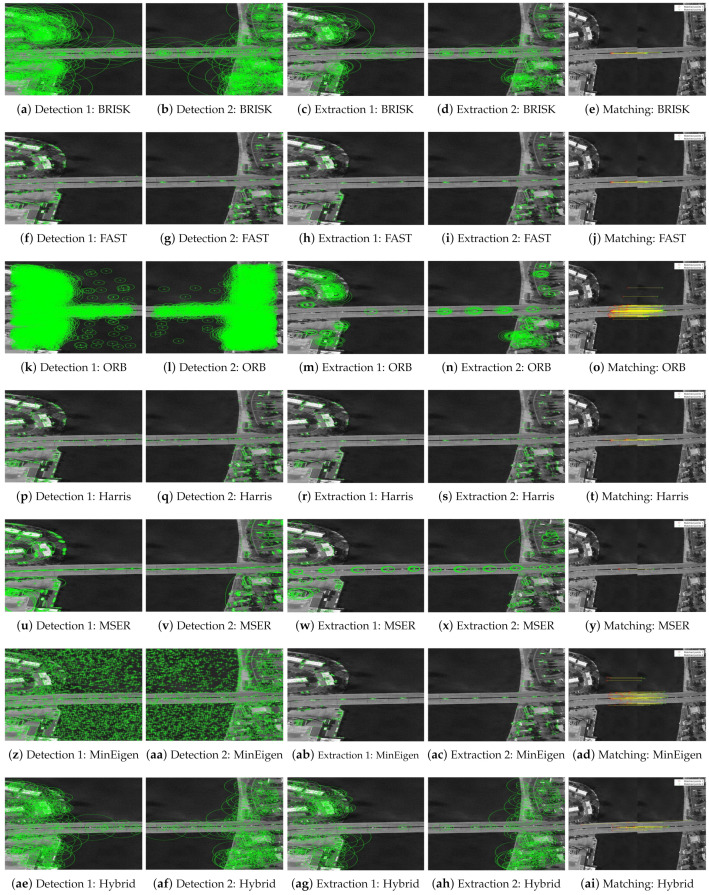
Sequential presentation of detection, extraction, and matching phases for various feature detectors on two sets of bridge aerial images. Each row represents a different detector and showcases the process from detection to matching.

Figure 13 and Figure 14 illustrate the process of feature point detection, extraction, and matching for these aerial images. Each figure sequences through the stages of detecting features in individual images, extracting those features, and then matching them between two images of the same scene. This sequence is demonstrated using different feature detectors and descriptors, highlighting how each algorithm performs under the same conditions.

The quantitative analysis of these experiments is presented in Table 5, which details the performance of each detection method across various metrics such as the number of keypoints detected, extracted, and successfully matched, as well as the efficiency metrics including match rate percentage and execution times (elapsed, CPU, and PMT). For instance, when comparing the two instances of the airport images, the hybrid algorithm significantly outperformed other methods with a matching rate of 73.90%, which is the highest among all the detectors. This high performance is consistent across the different images, underscoring the hybrid algorithm’s robustness and efficiency, particularly noted by its minimal processing time.

The results for the bridge images show similar trends, where the hybrid algorithm again demonstrates superior performance, especially in terms of execution time, making it an ideal candidate for real-time applications in remote sensing and aerial reconnaissance.

Overall, the scene-to-model registration experiments validate the efficacy of the proposed hybrid algorithm, not only in achieving high match rates but also in maintaining lower computational costs, making it suitable for real-time image analysis applications.

### 4.4. Scaling Transformations with Differet Scale Vectors

This subsection explores the effects of scaling transformations on feature detection and matching using three different feature detectors: BRISK, MSER, and Hybrid. The choice of BRISK and MSER stems from their known scale–space invariant properties, making them ideal for studying scaling impacts. The Hybrid detector is introduced to assess potential improvements in scaling performance.

#### 4.4.1. Comparative Analysis of Feature Detectors

The analysis is supported by three detailed tables that compare the performance of these detectors under different scaling vectors on two distinct images—the VSSUT entrance and the Hirakud dam. These tables (Table 6, Table 7 and Table 8) quantify the effects of scaling on detection accuracy and computational efficiency, essential for understanding the scalability of each detector.

The comprehensive performance comparison facilitated by Table 6, Table 7 and Table 8 provides a robust basis to evaluate the effectiveness of the BRISK, MSER, and Hybrid feature detectors under various scaling transformations. These tables incorporate crucial image quality metrics such as PSNR (Peak Signal-to-Noise Ratio) and MSE (Mean Squared Error) along with essential time-based metrics including elapsed time, CPU time, and PMT (Preprocessing and Matching Time). This extensive dataset not only validates the efficiency of the feature-detection algorithms but also highlights the computational demands associated with each method.

A consistent pattern is observed in the data, which underscores the superior performance of the Hybrid detector in maintaining high image quality metrics and managing computational time effectively across different scaling scenarios. For example, under a scaling vector of 2.0, the Hybrid detector consistently demonstrates higher PSNR values and lower MSE, indicating better image reconstruction with fewer errors compared to the BRISK and MSER methods. This efficiency is also reflected in the processing times, where the Hybrid detector often equals or surpasses the speed of the other detectors while delivering more accurate results.

This evaluation emphasizes the importance of selecting an appropriate feature-detection strategy based on specific application requirements, especially in environments that involve significant variations in image scale. The Hybrid detector, with its robust performance across various scales, emerges as an exceptionally effective tool in scenarios where both precision and efficiency are crucial. It proves to be an excellent choice for applications such as aerial imaging, surveillance, and other forms of remote sensing where images may undergo various transformations and require high fidelity and rapid processing for timely decision-making.

#### 4.4.2. Impact of Scaling on Feature Detection

**Figure 15:**
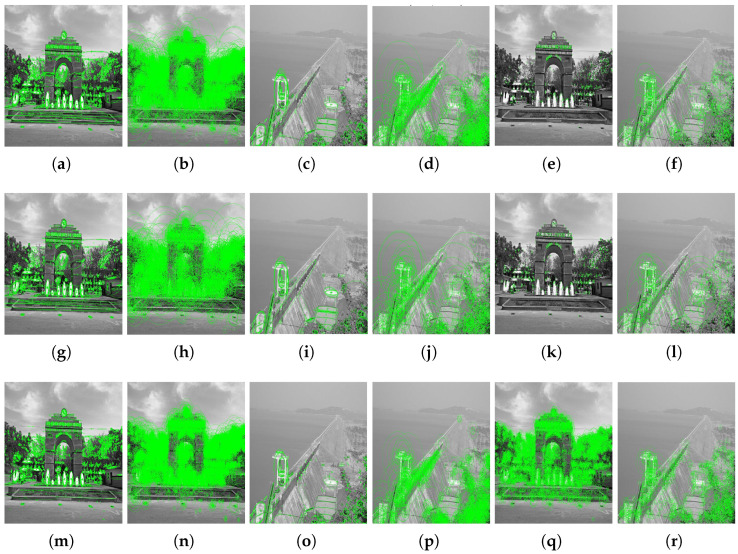
Comparison of feature-detection performance using MSER, BRISK, and Hybrid detectors on two different images under scaling transformations. Each row demonstrates the response of the detectors at scaling factors of the original, 0.7, and 2.0, highlighting the adaptability of these algorithms to changes in image scale. (**a**) MSER: VSSUT Gate Image. (**b**) BRISK: VSSUT Gate Image. (**c**) MSER: HD Image. (**d**) BRISK: HD Image. (**e**) Hybrid: VSSUT Gate Image. (**f**) Hybrid: HD Image. (**g**) MSER: VSSUT Gate Image, Scale 0.7. (**h**) BRISK: VSSUT Gate Image, Scale 0.7. (**i**) MSER: HD Image, Scale 0.7. (**j**) BRISK: HD Image, Scale 0.7. (**k**) Hybrid: VSSUT Gate Image, Scale 0.7. (**l**) Hybrid: HD Image, Scale 0.7. (**m**) MSER: VSSUT Gate Image, Scale 2.0. (**n**) BRISK: VSSUT Gate Image, Scale 2.0. (**o**) MSER: HD Image, Scale 2.0. (**p**) BRISK: HD Image, Scale 2.0. (**q**) Hybrid: VSSUT Gate Image, Scale 2.0. (**r**) Hybrid: HD Image, Scale 2.0.

**Figure 16:**
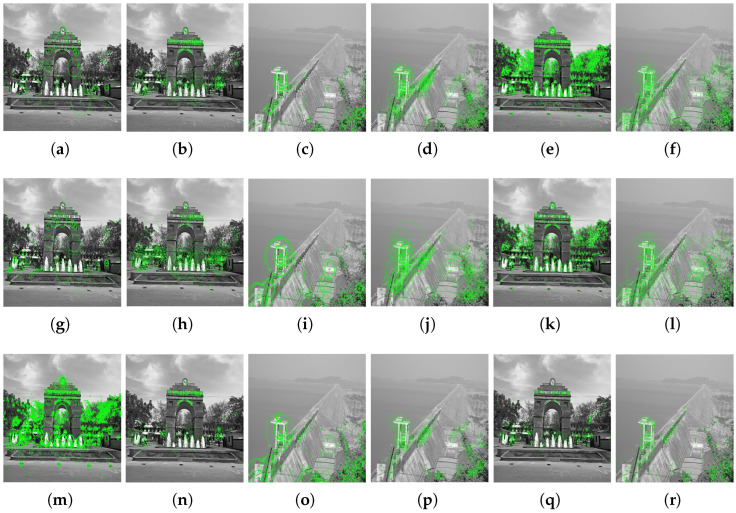
Extraction of feature keypoints using various extractors based on scaling factors of 0.7 and 2.0. Each row demonstrates the impact of scaling on the effectiveness of feature extraction across different images and detectors. (**a**) MSER: VSSUT Gate Image. (**b**) BRISK: VSSUT Gate Image. (**c**) MSER: HD Image. (**d**) BRISK: HD Image. (**e**) Hybrid: VSSUT Gate Image. (**f**) Hybrid: HD Image. (**g**) MSER: VSSUT Gate Image, Scale 0.7. (**h**) BRISK: VSSUT Gate Image, Scale 0.7. (**i**) MSER: HD Image, Scale 0.7. (**j**) BRISK: HD Image, Scale 0.7. (**k**) Hybrid: VSSUT Gate Image, Scale 0.7. (**l**) Hybrid: HD Image, Scale 0.7. (**m**) MSER: VSSUT Gate Image, Scale 2.0. (**n**) BRISK: VSSUT Gate Image, Scale 2.0. (**o**) MSER: HD Image, Scale 2.0. (**p**) BRISK: HD Image, Scale 2.0. (**q**) Hybrid: VSSUT Gate Image, Scale 2.0. (**r**) Hybrid: HD Image, Scale 2.0.

**Figure 17:**
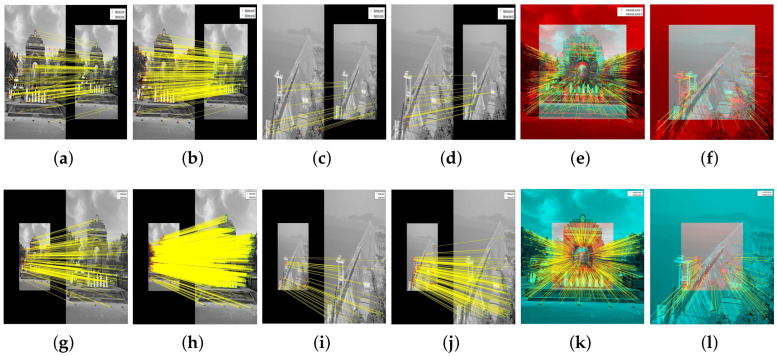
Matching of feature keypoints using various detectors on VSSUT gate and Hirakud dam images under two scaling factors, 0.7 and 2.0. Each image series demonstrates the effect of scaling on feature matching performance. (**a**) MSER: VSSUT Gate Image, Scale 0.7. (**b**) BRISK: VSSUT Gate Image, Scale 0.7. (**c**) MSER: Hirakud Dam Image, Scale 0.7. (**d**) BRISK: Hirakud Dam Image, Scale 0.7. (**e**) Hybrid: VSSUT Gate Image, Scale 0.7. (**f**) Hybrid: Hirakud Dam Image, Scale 0.7. (**g**) MSER: VSSUT Gate Image, Scale 2.0. (**h**) BRISK: VSSUT Gate Image, Scale 2.0. (**i**) MSER: Hirakud Dam Image, Scale 2.0. (**j**) BRISK: Hirakud Dam Image, Scale 2.0. (**k**) Hybrid: VSSUT Gate Image, Scale 2.0. (**l**) Hybrid: Hirakud Dam Image, Scale 2.0.

Figure 15, Figure 16 and Figure 17 demonstrate the efficacy of MSER, BRISK, and Hybrid feature detectors under varying scaling conditions, highlighting their capabilities and limitations in handling images from two distinctive scenarios: the VSSUT entrance and the Hirakud dam. Each image undergoes transformations using scaling vectors of 0.7 and 2.0, simulating conditions of both under-scaling and over-scaling, which are common challenges in practical applications.

In the initial set of images, MSER shows robustness in detecting features on the VSSUT gate under normal conditions, but its performance slightly degrades when the image is downscaled (0.7 scaling factor), suggesting a drop in sensitivity to smaller scale features. Conversely, BRISK maintains a consistent detection rate across scales, likely due to its design that balances scale invariance and feature stability. The Hybrid detector, expectedly, outperforms the individual MSER and BRISK in both scenarios by integrating their strengths and mitigating their weaknesses, particularly in the over-scaled (2.0 scaling factor) images where finer details are magnified.

Moving to the feature extraction figures, the pattern is somewhat consistent with the initial detection tests. The MSER detector captures denser clusters of keypoints in high-detail areas, which becomes sparse in scaled-down images. BRISK again shows uniform performance, proving to be less sensitive to scale changes compared to MSER. Hybrid’s advanced algorithm synergizes the detection process, ensuring that the quality and quantity of keypoints remain relatively stable across different scales. This adaptability makes it particularly suitable for applications requiring high precision in feature extraction across varied imaging conditions.

These observations underscore the importance of choosing the right feature-detection and extraction methods based on the specific requirements of the application, especially when dealing with images subjected to significant scale transformations. The Hybrid detector emerges as a strong candidate for scenarios requiring robust performance under diverse scaling conditions, providing a balanced solution that leverages the strengths of both MSER and BRISK.

#### 4.4.3. Advanced Analysis Using Registered Images

**Figure 18:**
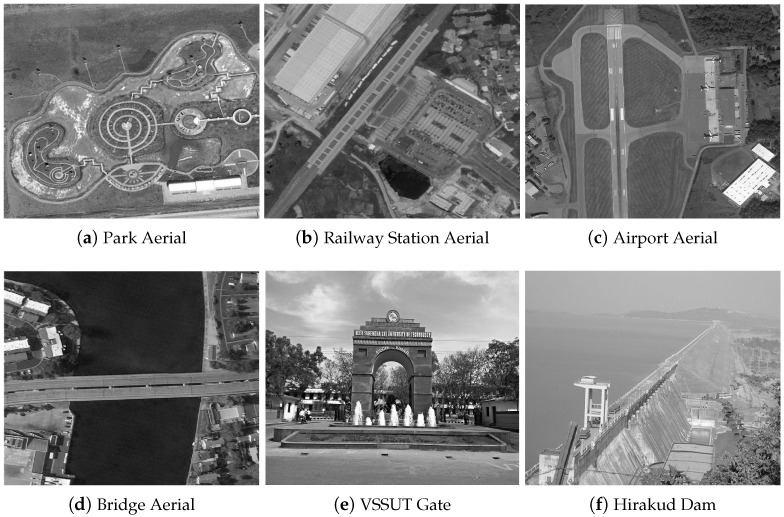
Registered images of different scenes using the Hybrid feature detector. Each subfigure shows a different aerial or scene image, highlighting the detailed synthesis achieved through the registration process.

Figure 18 displays the results of image registration using the Hybrid feature detector. This set of images showcases enhanced informational content through the integration of various scene details, achieving a more comprehensive representation of the original scenes. These registered images, processed through the Hybrid detector, exemplify the effectiveness of the method in synthesizing high-quality composite views from multiple aerial and terrestrial photographs.

**Figure 19:**
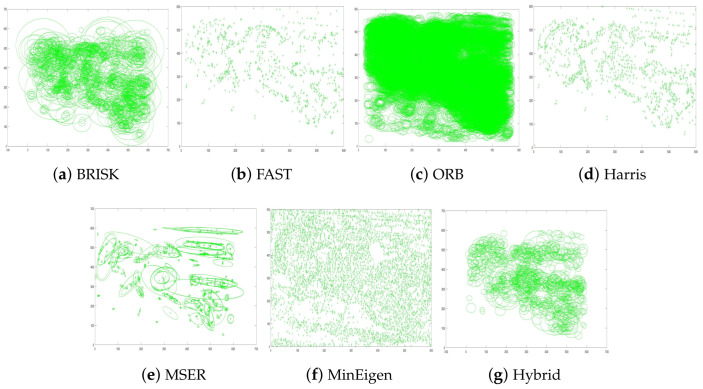
Performance comparison of various feature detectors on park scene images. Each subplot visually demonstrates how each feature detector identifies keypoints within the same environmental setting. This provides insights into the adaptability and precision of each method under similar conditions, highlighting their strengths and limitations in detecting significant image features effectively.

In Figure 19, each subplot visually demonstrates the keypoints detected by different feature-detection algorithms when applied to a park scene. These visual representations allow us to assess the density and distribution of keypoints identified by each algorithm, which are critical factors in evaluating their effectiveness and reliability for practical applications such as image registration and object recognition.

#### 4.4.4. Analysis of Feature-Detection Metrics

The performance of feature-detection algorithms is evaluated based on several key metrics, each offering insights into different aspects of the algorithm’s effectiveness. The Number of Keypoints detected is indicative of the algorithm’s ability to identify features across the image, which is crucial for comprehensive analysis and accurate matching. However, a larger number of keypoints does not necessarily equate to better performance, as the relevance and accuracy of these points are paramount.

Precision is a critical measure that assesses the accuracy and relevance of the detected keypoints. It measures the proportion of true positive keypoints among all detected keypoints, reflecting the accuracy of the detection process. The Matching Rate further complements these metrics by examining how effectively the keypoints from different images correlate, which is essential for applications like image stitching and 3D reconstruction. High matching rates suggest that the keypoints are not only accurately detected but are also meaningful in the context of aligning multiple images.

By evaluating these metrics together, we can form a comprehensive view of a detector’s performance, considering both the quantity and quality of the detected features. This approach ensures that the feature detectors are not only prolific in terms of keypoint generation but also precise and practical for real-world applications.

### 4.5. Discussion

This study’s comprehensive evaluation highlights the enhanced capabilities of our novel hybrid feature-detection algorithm within the context of Feature-Based Image Registration (FBIR). By integrating the strengths of established detectors such as BRISK, FAST, ORB, Harris, MinEigen, and MSER, the hybrid detector excels in both accuracy and efficiency, especially notable in complex image transformations like rotation, scaling, and scene-to-model changes. Such enhancements are crucial for applications in remote sensing and automated surveillance, where precise and reliable feature matching is paramount.

The experimental results demonstrate a significant reduction in time complexity alongside improvements in the detection and matching of keypoints. These improvements are quantified through detailed performance metrics, underlining the hybrid detector’s robustness across varied operational scenarios. This robustness ensures that the hybrid approach is well-suited to the dynamic and often unpredictable environments typical of remote-sensing applications, which demand high levels of adaptability and precision.

Furthermore, the comprehensive statistical analysis and the enhanced performance metrics clearly justify the efficacy of the hybrid feature-detection method. This approach not only meets but exceeds the capabilities of traditional detectors, particularly in handling complex image transformations.

Despite the promising outcomes, this study also acknowledges inherent limitations linked to the hybrid detector’s complexity. The integration of multiple detection methods into a cohesive algorithm introduces challenges in balancing computational efficiency with detection efficacy. This balance is particularly delicate when considering the diverse and often conflicting characteristics of the individual detectors involved. For instance, while some detectors may excel in speed, others might offer greater accuracy, necessitating careful calibration and tuning to harness their collective strengths effectively.

Moreover, the performance of our hybrid detector, while superior, still depends on the quality and diversity of the input data. This dependency suggests that the hybrid system’s adaptability might be constrained by less variable datasets, potentially limiting its effectiveness in less-controlled environments. Addressing these challenges involves not only refining the algorithm’s architecture but also ensuring that it remains flexible and responsive to the evolving landscape of image registration technologies and methodologies.

## 5. Conclusions and Future Work

This study introduced a combined approach using a novel feature detector and descriptor, enhancing all four steps of Feature-Based Image Retrieval (FBIR). Our evaluations, focusing on metrics such as elapsed time, CPU time, and performance measurement time, demonstrate that the proposed hybrid detector surpasses existing state-of-the-art detectors in terms of both efficiency and accuracy.

The hybrid detector not only improves the accuracy of detecting feature keypoints but also significantly reduces time complexity compared to conventional methods. This makes it particularly valuable for real-time image processing applications where speed and accuracy are crucial.

Throughout the testing phase, which included three types of image transformations—rotation, scene-to-model, and scaling—the proposed detector consistently outperformed other detectors, delivering superior visual and numerical results while also reducing execution times. These findings confirm that the proposed feature detector is more efficient compared to existing feature detectors, making it a robust and effective tool for image-analysis tasks.

In future studies, we aim to extend the reach and effectiveness of our proposed hybrid feature detector. A primary focus will be on optimizing the algorithm to enhance its adaptability and performance under a broader range of image conditions and transformations. Additionally, integrating deep learning techniques may offer substantial improvements in feature-detection capabilities, particularly for complex image scenarios, thereby expanding the utility and accuracy of our approach. Testing the hybrid detector across various platforms and media types will also be crucial to thoroughly validate its effectiveness and robustness in diverse operational contexts.

Applying the detector in real-world scenarios such as surveillance, autonomous driving, and medical imaging is essential to assess its practical utility and operational efficiency. Moreover, exploring the performance of the hybrid detector on different hardware configurations could lead to optimizations for energy efficiency and processing speed, making it well-suited for use in embedded systems and mobile devices. Through these initiatives, we hope to refine the capabilities of the hybrid detector further and expand its applicability to meet the evolving challenges in digital image processing and analysis [8].

## Figures and Tables

**Table 1:** Summary of feature-detection and image-registration methods.

Method/Algorithm	Characteristics	Applications
SIFT [13]	Scale-invariant, robust to rotation	Multispectral image registration
MSER [38]	Stable regions, distinctive features	Text detection, multi-source matching
SURF [13]	Fast, robust to scale and rotation	Multispectral matching
BRISK [10]	Fast, scale and rotation invariant	Generic image registration
FAST [39]	Very fast, lacks rotational invariance	High-speed feature detection
ORB [10]	Combines FAST and BRIEF, with rotation invariance	Cost-effective real-time applications
Harris–Affine [11]	High precision in detecting corners, not scale-invariant	Corner detection in images
Multispectral Facial Recognition [12]	Incorporates visible and IR images using various detectors	Facial recognition across spectrums
HOG [14]	Histogram of Oriented Gradients for keypoint matching	Offline transformation models

**Table 2:** Comparison of different interpolation (INR) techniques.

Image Quality Parameters	INR Techniques	Transformation Types		
		Affine	Similarity	Projective
MSE	Nearest Neighbor	0.00438	0.00445	0.00431
	Bilinear	0.00285	0.00293	0.00286
	Bicubic	0.00214	0.00219	0.00221
RMSE	Nearest Neighbor	0.06620	0.06674	0.06565
	Bilinear	0.05335	0.05411	0.05348
	Bicubic	0.04626	0.04678	0.04704
SNR	Nearest Neighbor	18.28352	18.21146	18.35780
	Bilinear	20.16101	20.03561	20.18970
	Bicubic	21.39797	21.29897	21.24642
PSNR	Nearest Neighbor	23.58262	23.51229	23.65465
	Bilinear	25.45772	25.33521	25.48598
	Bicubic	26.69549	26.59826	26.79086

**Table 3:** Quantitative evaluation of feature-detection performance at various rotation angles for the railway station image. The table displays keypoint detection, extraction, and matching statistics for each detector.

Detector	Det. Kpts1	Det. Kpts2	Ext. Kpts1	Ext. Kpts2	Matched Kpts	Match Rate (%)	Elapsed Time (s)	CPU Time (s)	PMT Time (s)
Rotation Angle: $30^{\circ }$, Sum: 146.7800, Mean: 20.9685, Variance: 64.7572, Std. Dev.: 8.0471									
BRISK	572	736	430	680	72	10.59	11.42	12.84	11.42
FAST	234	291	207	288	54	18.75	4.71	4.19	4.71
MSER	678	591	678	591	78	13.20	6.79	8.56	6.79
ORB	6753	9936	6753	9936	3037	30.57	4.92	5.03	4.93
Harris	665	525	588	504	97	19.25	4.80	5.27	4.80
MinEigen	4140	3785	3573	3748	847	22.60	3.58	3.80	3.59
Hybrid	569	746	569	748	238	31.82	3.34	3.22	3.34
Rotation Angle: $70^{\circ }$, Sum: 149.5300, Mean: 21.3614, Variance: 48.5798, Std. Dev.: 6.9699									
BRISK	572	730	430	677	74	10.93	11.69	13.83	11.70
FAST	234	263	207	256	68	26.56	4.47	4.09	4.45
MSER	678	586	678	586	142	24.23	7.46	7.30	7.46
ORB	6753	9535	6753	9535	3073	32.23	5.01	4.70	5.02
Harris	665	479	588	450	75	16.67	3.48	3.11	3.49
MinEigen	4140	3640	3573	3593	701	19.51	3.26	3.03	3.27
Hybrid	569	732	569	732	142	19.40	2.86	2.44	2.85
Rotation Angle: $90^{\circ }$, Sum: 637.2900, Mean: 91.0414, Variance: 186.4632, Std. Dev.: 13.6551									
BRISK	572	569	430	426	268	62.91	3.99	3.78	4.00
FAST	234	234	207	207	205	99.03	3.95	3.70	3.95
MSER	678	678	678	678	678	100.00	5.89	5.44	5.89
ORB	6753	6753	6753	6753	6753	100.00	4.18	3.89	4.19
Harris	665	665	588	589	518	87.95	3.57	3.25	3.57
MinEigen	4140	4140	3573	3572	3122	87.40	2.91	2.34	2.91
Hybrid	569	569	569	569	569	100.00	2.71	2.41	2.71
Rotation Angle: $120^{\circ }$, Sum: 148.2700, Mean: 21.1814, Variance: 56.2275, Std. Dev.: 7.4985									
BRISK	572	716	430	663	92	13.88	3.49	3.64	3.49
FAST	234	291	207	288	49	17.01	4.68	4.47	4.68
MSER	673	591	678	591	78	13.20	7.73	8.45	7.73
ORB	6753	9936	6753	9936	3037	30.57	5.17	5.42	5.19
Harris	665	525	588	504	101	20.04	4.07	4.13	4.07
MinEigen	4140	3735	3573	3747	815	21.75	3.15	2.72	3.15
Hybrid	569	748	569	748	238	31.82	2.98	2.61	2.99
Rotation Angle: $150^{\circ }$, Sum: 150.1200, Mean: 21.4457, Variance: 45.5085, Std. Dev.: 6.7460									
BRISK	572	722	430	673	95	14.12	6.49	7.86	6.48
FAST	234	295	207	289	42	14.53	3.89	3.30	3.88
MSER	678	580	678	580	107	18.45	7.57	7.86	7.57
ORB	6753	10,381	6753	10,381	2964	28.55	5.10	4.91	5.08
Harris	665	471	588	451	84	18.63	3.42	3.30	3.42
MinEigen	4140	3424	3573	3388	838	24.73	3.48	3.19	3.48
Hybrid	569	736	569	736	229	31.11	2.88	3.19	2.88
Rotation Angle: $180^{\circ }$, Sum: 682.85, Mean: 97.5500, Variance: 33.1407, Std. Dev.: 5.7567									
BRISK	572	568	430	426	360	84.51	3.60	2.44	3.59
FAST	234	234	207	207	207	100.00	3.98	3.03	3.98
MSER	678	678	678	678	674	99.41	6.23	7.11	6.23
ORB	6753	6753	6753	6753	6753	100.00	3.75	3.75	3.75
Harris	665	665	588	588	585	99.49	3.64	3.27	3.63
MinEigen	4140	4140	3573	3568	3548	99.44	3.04	2.88	3.04
Hybrid	569	569	569	569	569	100.00	2.84	2.58	2.84

**Table 4:** Quantitative evaluation of feature-detection performance at various rotation angles for the park image. The table displays keypoint detection, extraction, and matching statistics for each detector.

Detector	Detected Kpts1	Detected Kpts2	Extracted Kpts1	Extracted Kpts2	Matched Kpts	Matched Rate (%)	Elapsed Time (s)	CPU Time (s)	PMT Time (s)
Rotation Angle: $30^{\circ }$									
BRISK	1634	1973	1499	1951	173	8.86	13.92	16.08	13.93
FAST	894	1128	859	1125	179	15.91	4.84	6.72	4.84
MSER	767	779	767	779	126	16.71	7.51	7.78	7.50
ORB	13,704	18,521	13,704	18,521	5177	27.95	7.45	10.48	7.44
Harris	1176	1081	1119	1049	162	15.44	4.61	4.13	4.60
MinEigen	5213	4590	4608	4550	645	14.17	4.35	3.89	4.34
Hybrid	976	1009	976	1009	290	28.74	3.63	3.59	3.64
Rotation Angle: $70^{\circ }$									
BRISK	1634	1906	1499	1872	177	9.45	13.29	13.25	13.29
FAST	894	951	859	944	170	18.00	4.88	5.11	4.88
MSER	767	739	767	739	179	24.22	6.74	7.88	6.74
ORB	13,704	17,713	13,704	17,713	5393	30.44	8.91	10.45	8.92
Harris	1176	1270	1119	1236	142	11.48	4.84	3.92	4.85
MinEigen	5213	4623	4608	4568	495	10.83	3.83	4.75	3.84
Hybrid	976	1063	976	1063	322	30.29	3.44	3.86	3.44
Rotation Angle: $90^{\circ }$									
BRISK	1634	1648	1499	1512	938	62.03	4.74	4.45	4.75
FAST	894	894	859	859	797	92.78	3.42	3.13	3.43
MSER	767	767	767	767	755	98.43	16.69	23.30	16.68
ORB	13,704	13,704	13,704	13,704	13,704	100.00	5.67	7.22	5.66
Harris	1176	1176	1119	1119	933	83.37	4.23	3.61	4.23
MinEigen	5213	5213	4608	4613	3600	78.04	3.64	3.72	3.64
Hybrid	976	976	976	976	976	100.00	2.55	2.77	2.55
Rotation Angle: $120^{\circ }$									
BRISK	1634	1972	1499	1948	156	8.00	4.31	4.72	4.31
FAST	894	1128	859	1125	185	16.44	3.61	4.41	3.61
MSER	767	779	767	779	126	16.17	6.58	7.91	6.59
ORB	13,704	18,521	13,704	18,521	5177	27.95	8.32	11.36	8.33
Harris	1176	1081	1119	1049	158	15.06	4.91	5.73	4.89
MinEigen	5213	4590	4608	4550	620	13.62	3.37	3.77	3.37
Hybrid	976	1009	976	1009	291	28.84	3.33	4.44	3.33
Rotation Angle: $150^{\circ }$									
BRISK	1634	1932	1499	1899	179	9.42	4.55	5.19	4.55
FAST	894	1144	859	1137	163	14.33	4.55	4.77	4.56
MSER	767	726	767	726	163	22.45	6.49	6.64	6.49
ORB	13,704	18,282	13,704	18,282	5132	28.07	7.57	11.28	7.57
Harris	1176	1210	1119	1182	149	12.60	4.01	4.11	4.02
MinEigen	5213	4632	4608	4592	559	12.17	3.73	3.77	3.73
Hybrid	976	1022	976	1022	267	26.12	3.61	3.71	3.61
Rotation Angle: $180^{\circ }$									
BRISK	1634	1634	1499	1501	1325	88.27	2.95	2.75	2.95
FAST	894	894	859	861	859	99.76	4.06	3.00	4.06
MSER	767	767	767	767	754	98.30	6.78	6.33	6.79
ORB	13,704	13,704	13,704	13,704	13,704	100.00	6.56	7.88	6.56
Harris	1176	1176	1119	1121	1118	99.73	4.24	3.22	4.24
MinEigen	5213	5213	4608	4615	4590	99.45	3.51	2.73	3.50
Hybrid	976	976	976	976	976	100.00	2.81	2.94	2.81

**Table 5:** Scene-to-model registration, i.e., different images of the same scene applied on two sets of aerial images: airport and bridge.

Detection Method	Detected Kpts1	Detected Kpts2	Extracted Kpts1	Extracted Kpts2	Matched Kpts	Matched Rate (%)	Elapsed Time	CPU Time	PMT Time
Airport Aerial Images									
BRISK	278	731	195	604	24	19.85	4.93	4.73	4.93
FAST	201	464	150	404	28	34.65	6.11	5.25	6.12
MSER	173	270	173	270	34	12.59	6.26	5.30	6.25
ORB	1253	3759	1253	3759	129	17.15	5.51	4.56	5.51
Harris	153	342	117	289	21	36.30	5.52	4.83	5.52
MinEigen	955	2176	697	1689	100	29.60	5.59	4.09	5.59
Hybrid	89	257	89	257	38	73.90	4.48	3.86	4.47
Bridge Aerial Images									
BRISK	830	577	644	412	7	8.45	5.69	4.69	5.68
FAST	475	294	397	239	9	18.80	4.53	4.14	4.53
MSER	558	385	558	385	7	9.05	6.80	6.98	6.80
ORB	3805	3573	3805	3573	126	17.60	5.08	5.02	5.08
Harris	435	382	350	329	12	18.20	5.11	4.64	5.13
MinEigen	3664	3465	3101	2897	48	8.25	4.94	4.95	4.94
Hybrid	367	282	367	282	14	24.80	4.45	4.54	4.45

**Table 6:** Different sizes, i.e., scaling vectors applied on VSSUT entrance and Hirakud dam images using the BRISK detection method.

Image Name	Scaling Vector	Scaled Size	IQA	Bicubic	Bilinear	Nearest
VSSUT	0.7	717 × 538	PSNR	30.31	29.52	26.74
1024 × 768 134 KB		65.7 KB	MSE	0.00093	0.00112	0.00212
Hirakud dam	0.7	385 × 289	PSNR	31.75	29.33	26.70
550 × 412 34.7 KB		15.4 KB	MSE	0.00067	0.00117	0.00214
VSSUT	2.0	2048 × 1536	PSNR	26.60	25.94	24.38
1024 × 768 134 KB		330 KB	MSE	0.00219	0.00249	0.00364
Hirakud dam	2.0	1100 × 824	PSNR	31.31	30.20	28.81
550 × 412 34.7 KB		73.2 KB	MSE	0.00074	0.00095	0.00131

**Table 7:** Different sizes, i.e., scaling vectors applied on VSSUT entrance and Hirakud dam images using the MSER detection method.

Image Name	Scaling Vector	Scaled Size	IQA	Bicubic	Bilinear	Nearest
VSSUT	0.7	717 × 538	PSNR	30.66	30.31	26.59
1024 × 768 134 KB		65.7 KB	MSE	0.00086	0.00093	0.00219
Hirakud dam	0.7	385 × 289	PSNR	29.83	29.14	25.68
550 × 412 34.7 KB		15.4 KB	MSE	0.00104	0.00122	0.00270
VSSUT	2.0	2048 × 1536	PSNR	26.87	25.92	24.21
1024 × 768 134 KB		330 KB	MSE	0.00206	0.00256	0.00379
Hirakud dam	2.0	1100 × 824	PSNR	30.57	28.03	25.47
550 × 412 34.7 KB		73.2 KB	MSE	0.00088	0.00157	0.00283

**Table 8:** Different sizes, i.e., scaling vectors applied on VSSUT entrance and Hirakud dam images using the Hybrid detection method.

Image Name	Scaling Vector	Scaled Size	IQA	Bicubic	Bilinear	Nearest
VSSUT	0.7	717 × 538	PSNR	31.47	30.34	27.02
1024 × 768 134 KB		65.7 KB	MSE	0.00071	0.00093	0.00198
Hirakud dam	0.7	385 × 289	PSNR	34.11	31.66	26.78
550 × 412 34.7 KB		15.4 KB	MSE	0.00039	0.00068	0.00210
VSSUT	2.0	2048 × 1536	PSNR	26.89	26.04	24.38
1024 × 768 134 KB		330 KB	MSE	0.00205	0.00249	0.00364
Hirakud dam	2.0	1100 × 824	PSNR	31.31	29.75	25.93
550 × 412 34.7 KB		73.2 KB	MSE	0.00074	0.00106	0.00255

## Data Availability

Experimental aerial images were obtained from the following repository: https://captain-whu.github.io/AID/, (accessed on 29 August 2024).
